# 

**Published:** 1891-02

**Authors:** 


### TABLE OF CONTENTS.

**ORIGINAL COMMUNICATIONS.** PAGE

Inter-Dental Splints. By ALONZO P. BEALE, D.D.S., Philadelphia . . . 81

Erosion. By WILLIAM H. TRUEMAN, D.D.S., Philadelphia . . . . . . 86

Why has the American Dentist a High Reputation throughout the Civilized World? By DR. C. T. TERRY, Milan, Italy . . . . . . . . . . 97

Independent Dental Journalism. By L. ASHLEY FAUGHT, D.D.S., Philadelphia . . . . . . . . . . . . . . . . . . . . . . . . 100

A Case in Practice. By J. G. W. WERNER, D.M.D. . . . . . . . . . 105

**REPORTS OF SOCIETY MEETINGS.**

American Dental Association.—Thirtieth Annual Meeting, Excelsior Springs, Mo., August 5 to 8, 1890 . . . . . . . . . . . . . . . . . 109

New Jersey State Dental Society.—Twentieth Annual Session . . . . . . 117

Pennsylvania State Dental Society . . . . . . . . . . . . . . . . . 125

Swiss Odontological Society . . . . . . . . . . . . . . . . . . . . 127

**EDITORIAL.**

The Tariff on Manufactured Teeth . . . . . . . . . . . . . . . . . 130

Dr. (?) Hunter's Dental Anodyne . . . . . . . . . . . . . . . . . . 131

To Contributors . . . . . . . . . . . . . . . . . . . . . . . . . . 132

Bibliography . . . . . . . . . . . . . . . . . . . . . . . . . . . 132

**DOMESTIC CORRESPONDENCE** . . . . . . . . . . . . . . . . . . . 138

**CURRENT NEWS** . . . . . . . . . . . . . . . . . . . . . . . . . 142

